# A Management Dilemma: Infectious Keratitis Associated with Soft Contact Lens Use and Dubious Treatment Compliance

**DOI:** 10.1155/2010/415737

**Published:** 2010-08-10

**Authors:** Konstantinos T. Tsaousis, Georgios Sakkias, Nikolaos Kozeis, Periklis Tahiaos

**Affiliations:** Department of Ophthalmology, “Hippokration” General Hospital, 54642 Thessaloniki, Greece

## Abstract

*Purpose*. To present a case of infectious keratitis caused by the microorganism Serratia marcescens in a contact lens user and further to confer on the most advantageous management of comparable situations. *Case*. After altering the routine that she used for contact lens disinfection, a 24-year-old patient presented with pain and conjunctival redness in both eyes. Slit-lamp examination revealed two infiltrates in the inferior part of the cornea in the right eye and five smaller infiltrates in the superior half of the left cornea. Appropriate treatment, after hospitalization, improved the symptoms while culture of the contact lens material revealed Serratia marcescens as the responsible infectious factor. *Conclusion*. Enhancing the availability of information with respect to contact lens users and customized analysis regarding treatment for a particular complication could be beneficial in order to reduce the frequency of admission to the eye clinic due to infectious keratitis. In addition, rapid laboratory testing of the infected materials should be a priority for selection of the optimal treatment regimen.

## 1. Background

Serratia species are opportunistic Gram-negative bacteria classified in the tribe Klebsielleae and the large family Enterobacteriaceae. The primary pathogenic species is Serratia marcescens [1].

Serratia marcescens is a potential cause of infectious keratitis that appears to be associated with abnormal corneal surface, topical medications, and contact lens wear. Proper medical treatment results in a good clinical response in most cases [2, 3].

Current guidelines [4] suggest that admission to the hospital because of keratitis may be necessary if

- the infection is sight-threatening,
- the patient has difficulty administering the antibiotics at the prescribed frequency,
- there is high likelihood of noncompliance with drops or daily followup
- suspected topical anaesthetic abuse,
- intravenous antibiotics are needed (e.g., corneal perforation, scleral extension of the infection, and gonococcal conjunctivitis with corneal involvement).

Serratia endophthalmitis usually occurs after ocular surgery with poor prognosis [5–7].

## 2. Case Report

A 24-year-old female patient presented to the emergency department complaining of severe pain and redness in both eyes.

The patient had a clear medical record. She mentioned that she is a regular contact lens user due to myopia and had never experienced problems with her eyes. She had been wearing soft contact lenses for the last six years on a daily wear basis for eight hours per day, seven days per week and used OptiFree Express (Alcon, Fort Worth, Texas) as a disinfecting solution. She claimed to never wear her contact lenses overnight. The patient also mentioned that she had recently switched to a different contact lens disinfecting solution. She started to use Renu fresh multipurpose solution (Bausch & Lomb, Rochester, New York) based on the recommendation of her optician. Finally she mentioned that her lenses, suitable for use for one month, were not used for more than one week, and that the pair currently in use were in fine condition.

Best corrected visual acuity was 20/20 on both eyes despite the fact that a sense of blurred vision was reported.

Slit-lamp examination revealed two infiltrates, approximately 1,5 mm in size, at 4 and 7 o'clock in the inferior part of the cornea in the right eye and five smaller infiltrates in the superior half of the left cornea. The infiltrates appeared to extend to the middle stroma. Furthermore, conjunctival redness and moderate anterior chamber inflammation were observed in both eyes.

Regarding management, the appropriate guidelines were applied [8].

Immediately, prior to any action, the infected contact lenses and a specimen of the disinfecting solution were taken and sent in for culture [9].

Initially, although the patient was informed of her condition and advised to follow the appropriate treatment in her own house, she demonstrated significant anxiety about the way she should administer the medication and subsequently was advised to remain in the clinic for treatment.

The treatment regimen included Ofloxacin drops 0.3%, fortified Gentamycine drops (14 mg/ml), topical use of diluted Povidone Iodine solution, and Dexpanthenol eye gel.

After 24 hours the ocular symptoms improved significantly, and after 48 hours the patient decided to continue the treatment in her home.

The patient returned two days later and slit-lamp examination revealed healing of the left eye lesions and improvement of the infiltrates in the right eye. The results of the culture demonstrated that the responsible microorganism was Serratia Marcescens, which is sensitive to several antibiotics. The patient was encouraged to continue the use of Tobramycin drops and ointment only. Moreover, she was advised to return for a final check after five days. This visit eventually confirmed complete elimination of the symptoms. 

The study on the case was accomplished according to the tenets of the Declaration of Helsinki, and the patient provided her informed consent after the nature and intent of the study had been fully explained to her [10].

## 3. Discussion

In contact lens wearers, keratitis represents a perturbed condition. Its incidence can be reduced by maintaining high standards in terms of lens hygiene and generally following the recommended guidelines [11–13].

In this particular case, there was not much complexity with regard to the course of treatment prescribed but rather issues had to be resolved in terms of where the treatment would take place.

Although the patient was referred due to problems associated with contact lens use, she expressed noteworthy frustration when she was forced to face one of them. She presented denial and anger at the time of the initial diagnosis and a manner of depression immediately afterwards. This reaction precipitated her admission to the clinic. After a detailed discussion with the doctor she finally accepted her condition but even then she preferred to stay in the hospital reporting that she considered herself unable to follow the therapeutic instructions at home. A feeling that is customary in a considerable amount of analogous situations [14, 15]. 

 The incident made clear to the medical personnel that there is a dire need that more detailed information must be provided to contact lenses users concerning the prevention and management of potential complications. 

A dilemma consequently emerges: relieving patient anxiety while unambiguously explaining the possible fallout in case of deficient treatment. This kind of caveat may engender two types of reactions: a feeling of conscientiousness and eagerness to follow the treatment regimen carefully or, in contrast, a panic attack that diminishes the ability of the patient to effectively administer self-treatment. It is vital to bear in mind that each patient reacts to hazards in an individual manner.

## 4. Conclusion

This case report confirms that the doctor must identify the unique needs of each person to maximize chances of the treatment's success [16]. Furthermore, identifying the precise cause of the infection can prevent unnecessary medical, financial, and ultimately emotional costs [17–20]. 

Efforts in this regard to minimize the time required for the identification of the microorganisms that cause infectious keratitis are currently underway [21–23]. Progress in this area is crucial for the management of situations similar to the one described, briefly, in the present study.
